# Bark anatomy of *Euphorbia tirucalli* (Euphorbiaceae): a unique way of bark dilatation on a stem succulent tree

**DOI:** 10.1093/aobpla/plaf007

**Published:** 2025-02-12

**Authors:** Kolwane Calphonia Shilaluke, Annah Moteetee, Alexei Oskolski

**Affiliations:** Department of Botany and Plant Biotechnology, University of Johannesburg, P.O Box 524, Auckland Park, 2006 Johannesburg, South Africa; Department of Botany and Plant Biotechnology, University of Johannesburg, P.O Box 524, Auckland Park, 2006 Johannesburg, South Africa; Department of Botany and Plant Biotechnology, University of Johannesburg, P.O Box 524, Auckland Park, 2006 Johannesburg, South Africa; Komarov Botanical Institute, Prof. Popov Street 2, 197376 St. Petersburg, Russia

**Keywords:** photosynthesis, phellogen initiation, periderm, expansion cracks, stomatal grooves, stomata, fibre-sclereids

## Abstract

The covering tissue structure is important for leafless stem succulents, influencing their photosynthetic activity. Usually, the epidermis on succulent stems persists for a long time, making the stem photosynthesize as long as possible. Its persistence implies maintaining the continuity of covering tissues during bark dilatation. In most plants, dilatation is performed by developing periderm(s) to replace the epidermis. The ways of bark dilatation with delay of periderm formation remain poorly known. In the present study, we examine the structure of the juvenile and mature bark of succulent pencil tree *Euphorbia tirucalli* (Euphorbiaceae) to clarify the ways of dilatation of its protective tissues. The bark structure of *Euphorbia tirucalli* at various developmental stages was examined using light and scanning electron microscopy. The epidermis is found not only on young stems of *E. tirucalli* but also on the bark of its thick branches and trunk, which are up to about 6 cm in diameter. On the young stems, the stomata are sunken in long vertical grooves. On the older stems, these grooves are stretched out due to dilatation, bringing stomata to the surface. Expansion cracks, i.e. broad vertical epidermal ruptures underlaid by tightly arranged tangential strands of cortical parenchyma, appear between the stomatal zones on dilated bark. The phellogen is initiated in the depth of the cortex beneath the epidermal ruptures long after their formation. The bark dilatation by forming epidermal ruptures with delayed initiation of periderm was found for the first time. Prolonged photosynthesis in the succulent stem is the main functional benefit of such a bark dilatation method. The initiation of periderm in the depth of the cortex has not been reported in stem succulents other than a few *Euphorbia* species.

## Teaser

The stem of *Euphorbia tirucalli* , a succulent plant, undergoes remarkable structural changes after leaf shedding. Stomata, essential for gas exchange, are positioned within deep stomatal grooves that facilitate the movement of carbon dioxide into the underlying tissue (cortical parenchyma). As the stem expands, these stomatal grooves stretch, eventually bringing the stomata to the surface. Unlike most plants, where bark expansion is driven by periderm growth, the epidermis in *E. tirucalli*  remains intact on thick branches and trunks but expands through the formation of expansion cracks rather than uniform stretching. These ruptures are underlaid by densely packed tangential strands of cortical tissue beneath them. Interestingly, the periderm develops only after these cracks form, a process not previously documented in other plants. Additionally, the formation of the phellogen (a layer responsible for producing protective bark tissue) occurs unusually deep in the cortex, an uncommon trait among *Euphorbia* species and other stem succulents. The study also reclassifies sclerenchyma cells in the bark as fibre-sclereids rather than traditional fibres, refining our understanding of this plant’s anatomy.

## Introduction

*Euphorbia* is the most species-rich genus of the family Euphorbiaceae and one of the largest genera of flowering plants. This nearly cosmopolitan genus comprises about 2000 species showing tremendous morphological diversity. Habit in *Euphorbia* ranges from small, annual herbs to large trees, including diverse forms of succulents with water-storing leaves, aerial stems of different (cacti-like, pencil-stemmed, medusoid, etc.) appearance, or below-ground caudices and tubers (Horn *et al.* 2012, Evans *et al.* 2014, Webster 2014). Despite the variability of the vegetative body, all species of *Euphorbia* share a unique inflorescence type, the cyathium, that is, a pseudanthium (false flower) evolved from a thyrse (Prenner and Rudall 2007, Prenner *et al.* 2008). The presence of non-articulate laticifers producing white milk-like latex is another conspicuous feature of *Euphorbia* (Rudall, 1987).

The great diversity of xeromorphic growth forms in *Euphorbia* arose from numerous independent evolutionary shifts to stem succulency (Horn *et al.* 2012). Apart from extreme parallelism in habit evolution within the genus, *Euphorbia* shows many remarkable convergent similarities between habit and vegetative organs with Cactaceae and stem succulents from other plant families (e.g. Mauseth 2004). Such parallelisms provide excellent opportunities to clarify the evolution of structural and functional traits associated with stem succulency. While the family Cactaceae serves now as a model group in the studies of structural diversity and evolution of this habit (e.g. Mauseth 2006, 2017a, 2017b, 2021, Guerrero *et al.* 2019), the anatomy and morphology of stem succulents in *Euphorbia* remain much less studied (e.g. Mauseth 2004, Kalashnik and Gaidarzhy 2013, Kalashnik 2015, Arévalo-Rodrigues *et al.* 2022; etc.). Anatomical exploration of *Euphorbia* species with comparative analysis of their traits with those in Cactaceae and other plant groups can provide valuable insights into the structural background of the evolution of stem succulency.

The structure of covering tissues is of great functional importance for succulents, influencing the allocation of their photosynthetic activity. Usually, the epidermis of stem succulents persists for long periods (up to decades or even about 200 years), as has been reported for the columnar cactus *Cereus giganteus* Engelm. (Darling 1989), making stem photosynthesis possible (Mauseth 2004, 2006). A similar condition is also known in some non-succulent plants, such as *Parkinsonia procera* (Ruiz & Pav.) Hawkins (Fabaceae): the photosynthetic stems of this tree are mostly covered by multilayered tissue derived from a meristematic zone of epidermal origin termed as ‘special epidermis’ (Aguilar-Rodriguez *et al.* 2019). The periderm in such plants forms only on older parts of stems (Gibson and Nobel 1986, Mauseth 2006, Hearn 2009, Garcia *et al.* 2012) and in response to traumatic effects (Evans and Scelsa 2014, Evans and Cooney 2015). The early formation of periderm also occurs in some stem succulents, as in caudiciform shrubs of *Euphorbia* (Horn *et al*., 2012). Although the presence of periderm does not exclude the stem photosynthesis in the stems of leaf-bearing trees (Nilsen 1995, Kotina *et al.* 2012, 2017, Guía-Ramírez *et al.* 2020), its hampering effects on access to light and gas exchange could be crucial for leafless plants. Thus, the anatomical details of periderms, the localization, and the timing of their initiation are of great interest in that respect.

The persistence of the epidermis on the surface of thick stems implies the ability of this tissue and its underlaid cortex to maintain its continuity during bark dilatation (tangential stretching due to the stem growth in girth). In most plants, the outer bark is dilated by forming periderm or multiple periderms, replacing the epidermis already on young stems (Angyalossy *et al.* 2016). The transformations of dilated barks lacking periderm remain very poorly known.

In the present study, we examine the structure of the juvenile and mature bark of the pencil tree *Euphorbia tirucalli* L., emphasizing its changes during dilatation and the formation of its periderm. Although bark anatomical information is available for many species of *Euphorbia* (Rao *et al*., 1968; Mauseth, 2004; Schweingruber and Landolt, 2005; Thakur and Patil, 2012; Kalashnik and Gaidarzhy, 2013; Crivellaro *et al*., 2013; Kalashnik, 2015; Lekshmi *et al.*, 2017; Sultana 2017; Anandan *et al*., 2018; Acharya *et al*., 2021; James *et al*., 2022; Arévalo-Rodrigues *et al*., 2022), the published data are mostly based on examination of young stems. Some anatomical characteristics of mature bark in several *Euphorbia* species have been reported by Zahur (1959) and Mtombeni (1994), but these authors did not consider important details of their dilatation and periderm development, which are the points of our interest.

*Euphorbia tirucalli* is an unarmed shrub or branched tree 2–5 m tall (occasionally up to 10 m) with finger-like succulent shoots of 10–25 cm in length. Young shoots bear a few small, slender leaves which fall off early. Branches and thin trunks of *E. tirucalli* have green smooth bark, replaced by rough bark in their thicker portions. When injured, all parts of these plants produce abundant milky latex (Baniakina and Eyme 1997, Coates Palgrave 2002, Rojas-Sandoval 2016). The species is widespread in tropical and subtropical regions of all continents, but its natural distribution is unclear: Africa and India are considered possible regions of origin (Rojas-Sandroval 2018). The latex of *E. tirucalli* is used traditionally to treat various ailments such as spleen infections, colic pains, asthma, rheumatism, earache, cough, and toothache (Priya and Rao 2011). This latex has also been recorded to have insecticidal properties against insects such as aphids and moths (Priya and Rao 2011, Mwine *et al.* 2013, Roy *et al.* 2020). Apart from that, *E. tirucalli* is considered a prospective source of biofuel (Nchimbi 2021).

The data on the anatomical structure of leaves and/or young stems of *E. tirucalli* has been reported by Rao *et al.* (1968), Kalashnik and Gaidarzhy (2013), Kalashnik (2015), Lekshmi *et al.* (2017) and Anandan *et al.* (2018). Wood anatomy of this species has been studied by Mennega (2005), Jangid *et al.* (2017), and Siegloch (2014); a brief anatomical description of its mature bark was provided by Mtombeni (1994). In the present study, we revealed some prominent changes in the epidermis associated with the growth of photosynthetic stems in girth, including the appearance of stomatal grooves with their subsequent unfolding, as well as the appearance of expansion cracks with conspicuous epidermal ruptures formed long before the initiation of phellogen. These cracks have never been described on the bark of mature stems in other plant taxa; their formation represents a poorly known way of dilatation, which is thought to be found in photosynthetic barks of other stem succulents as well as in the bark of young stems of other woody plants.

## Materials and Methods

**Figure 1.**
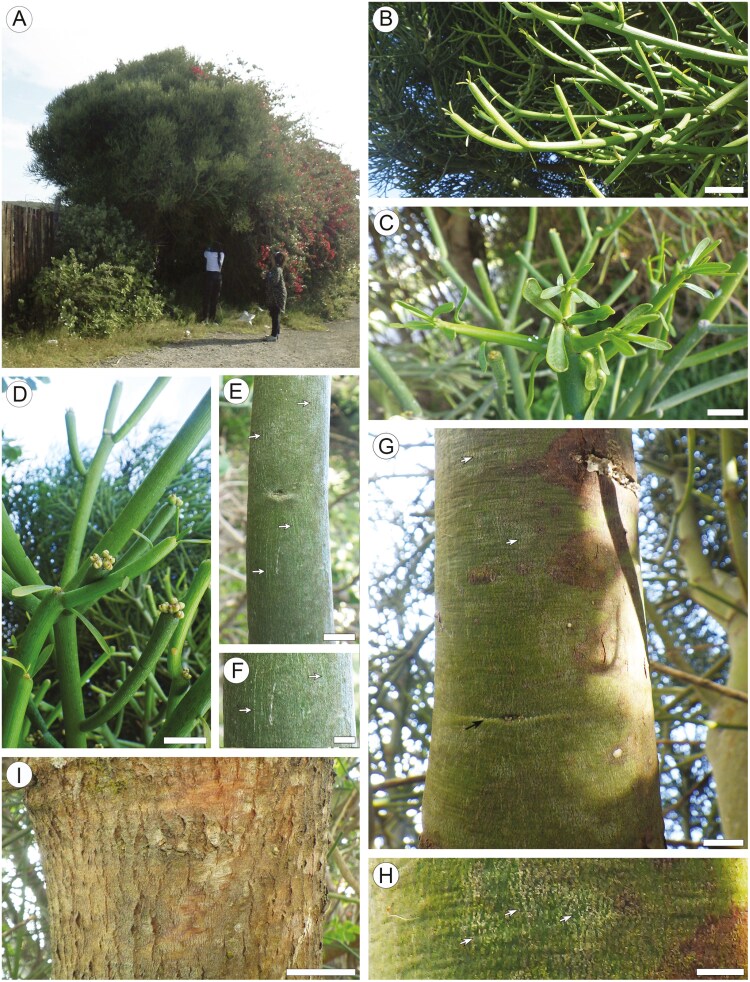
Gross morphology of the studied individual of *Euphorbia tirucalli*. (a) Entire tree. (b), (c) Juvenile shoots with leaves. (d) Older leafless shoots, clusters of cyathia on their tips. (e, f) Surface of a twig bearing leafless shoots with stomatal grooves appearing as whitish vertical striation (arrows). (g, h) Surface of trunk (6 cm in diameter) with smooth mature bark bearing expansion cracks appearing as vertical whitish flecks (arrows) and eye marking. (i) Surface of trunk (21 cm in diameter) bearing corky mature bark with shallow fissures. Scale bars: a = 2 m; b–d = 10 mm; e = 5 mm; f and g = 50 mm.

The samples of *Euphorbia tirucalli* [A. Oskolski # 697-22, abbreviated below as AO 697-22] used for the anatomical investigations was collected by the authors on 28.11.2022 from the tree (Fig. 1a) cultivated near road R72 near Fort d’Acre, Eastern Cape province, South Africa. The samples used for quantitative assessment of bark growth ([AO 768-24], [AO 770-24]) were collected by the first and third authors on 28.06.2024 from the shrubs cultivated near the Auckland Park Kingsway Campus of the University of Johannesburg, Johannesburg, Gauteng province, South Africa. The shoots, bark, and wood pieces were stored and fixed in 70% ethanol until use. A voucher specimen (AO 697-22) was deposited at the University of Johannesburg Herbarium (JRAU).

For anatomical examination of juvenile stems and mature bark, transverse and longitudinal sections (radial and tangential) of the bark were made with a freezing microtome (Ernst Leitz GMBH, Wetzlar, Germany). Sections were stained with a mixture of alcian blue/safranin and mounted in Euparal. For more detailed anatomical studies, the samples were also embedded in glycol methacrylate (GMA) according to a modification of the Feder and ‘O’Brien (1968) method. Transverse, tangential, and radial sections of about one μm thick were cut using a Porter Blum MT-1 98 ultramicrotome, then stained using the toluidine blue method and mounted in Entellan. Additionally, tangential sections of mature bark were sectioned with a freezing microtome (Ernst Leitz GmbH, Wetzlar, Germany) stained with a 1:1 alcian blue /safranin mixture and mounted in Euparal.

The details of the bark surface were also investigated using scanning electron microscopy (SEM, TESCAN, soft–VegaTS) at the Central Analytical Facility of the Faculty of Science, University of Johannesburg (Spectrum). Samples of bark for SEM observations were dehydrated in 96%, followed by 100% ethanol and isoamyl acetate. Dehydrated samples were critical-point dried using a Quorum Technologies Ltd (Ashford, Kent, England) HCP-2 critical-point dryer, mounted on aluminium stubs with double-sided carbon tape, and coated with gold using Emscope SC 500 series Coater Sputter for SEM.

The length of sieve tube members and fibre-sclereids was measured on the small fragments of secondary phloem macerated using Jeffrey’s solution (Johansen 1940). A test with vanillin-hydrochloric acid was used to detect tannins (Angyalossy *et al.* 2016). All the sections were studied with a light microscope (Olympus CX41RF). Sudan IV test (Johansen 1940) for lipids (including suberin) was used to detect the occurrence of lipids (including suberin) in the cells of cortex and periderm on handmade sections of fresh bark samples [AO 768-24]. Digital images were taken with a digital camera, an Olympus XC50, using an Olympus Stream Essentials 1.8 Imaging System. Descriptive terminology followed the recommendations of Angyalossy *et al.* (2016) for bark description.

Contributions of epidermal stretching and expansion cracks to the increase in the bark circumferences were assessed on the transverse sections (TS) of the stem portions of different diameters taken from the samples [AO 768-24] and [AO 770-24]. Four bark sections of ca. 25 µm thick were made from each stem portion on a freezing microtome (Ernst Leitz GMBH, Wetzlar, Germany) and mounted in glycerol without staining. Apart from that, the TS on permanent microslides of the sample [AO 697-22] were used for this purpose. The stem circumference as well as the tangential dimensions of the epidermis and expansion cracks, were estimated for each section, and the tangential size of 50 epidermal cells was measured for each stem portion. All measurements were performed under a light microscope Olympus CX41RF with the digital camera an Olympus XC50, using an Olympus Stream Essentials 1.8 Imaging System.

## Results

### Gross morphology of *Euphorbia tirucalli* (Fig. 1)

The bark samples for anatomical were collected from a branched tree of ca. 7 m tall [AO 697-22] (Fig. 1a). Additionally, the samples from two shrubs of 1.1 m tall [AO 768-24] and 1.7 m tall [AO 770-24] also bearing similar finger-like succulent shoots were taken for quantitative analysis of the increase in the circumference of smooth bark (Table 1). All three plants have finger-like succulent shoots of 8–22 cm in length (Fig. 1b and c) spirally arranged but clustered by the twig tips. The juvenile shoots (Fig. 1b and c) have a bright green surface and bear a few small, slender leaves 2–4 mm wide and 5–12 mm long. Older shoots are leafless (Fig. 1d), with a greyish-green [AO 697-22] or reddish to tangerine (AO 768-24; AO 770-24) surface having fine whitish vertical striation (Fig. 1e and f). Clusters of 3–8 preanthetic cyathia were found on the tips of some leafless shoots of [AO 697-22] (Fig. 1d). Smooth yellowish green bark (designated below as ‘smooth mature bark’) with whitish oval to diamond flecks found on older branches and trunks up to 4.5–6.0 cm in diameter (Fig. 1g and h). Eye marks occur on mature bark (Fig. 1g). Thicker stems bear brown bark (‘corky mature bark’) with shallow long fissures, without lenticels and any traits of scaling or peeling (Fig. 1i).

**Table 1.** Dimensions of epidermis, expansion cracks, and epidermal cells on the stems portions of different diameters in three samples of *E. tirucalli*.

Sample	Stem diameter, mm	Girth, mm	Epidermis: tangential dimension, mm (mean/min-max)	Expansion cracks: tangential dimension, mm (mean/min-max)	Expansion cracks: tangential dimension, % to the stem girth (mean/min-max)	Epidermal cells: tangential size, µm (mean /min-max)	Epidermis: relative increment of the tangential dimension (% of the preceding stem portion)	Epidermal cells: relative increments of the mean tangential size (% of the preceding stem portion)
AO 697-22	1.9	5.9	5.9	0	0	6.3±0.22 4.4–8.3	0	0
	3.1	9.7	9.7	0	0	6.7±0.22 2.9–11.4	63.1	7.4
	10.7	33.6	33.6	0	0	9.2±0.32 4.7–12.7	245.2	36.0
	12.5	39.2	39.2	0	0	9.4±0.44 5.9–16.4	16.7	2.5
	36.0	113.0	53.3 30.5–59.9	59.7 53.1–82.5	52.8 47.0–73.0	14.0±0.39 6.8–20.3	35.6	48.9
	45.2	141.9	61.8 48.7–71.9	80.1 70.0–93.2	56.4 49.3–65.7	16.9±0.42 11.3–26.0	15.9	20.5
AO 768-24	3.8	11.9	11.9	0	0	10.4±0.49 7.1–16.3	0	0
	6.5	20.4	20.4	0	0	12.3± 0.34 8.4–17.6	71.0	18.5
	11.5	36.1	36.1	0	0	12.8±0.32 8.5–18.3	76.9	3.8
	41.6	130.6	53.8 44.3–66.2	76.8 64.4–86.3	58.8 49.3–66.1	18.7±0.60 13.2–26.3	49.0	46.2
AO 770-24	2.9	9.1	9.1	0	0	7.1 ± 0.26 4.5–9.6	0	0
	6.1	19.1	19.1	0	0	10.7 ± 0.41 7.1–14.9	110.3	49.8
	13.5	42.4	42.4	0	0	11.6 ± 0.43 7.3–15.8	116.4	8.8
	34.0	106.8	54.1 45.2–62.1	52.7 44.7–61.6	49.3 41.8–57.7	15.5 ± 0.44 9.5–25.3	27.6	33.6
	61.2	192.2	68.0 54.0–82.6	124.2 109.6–138.2	64.6 57.0–71.9	19.2± 0.53 12.8–30.5	25.7	23.9

### Juvenile bark on leaf-bearing twigs (Fig. 2a–c)

**Figure 2.**
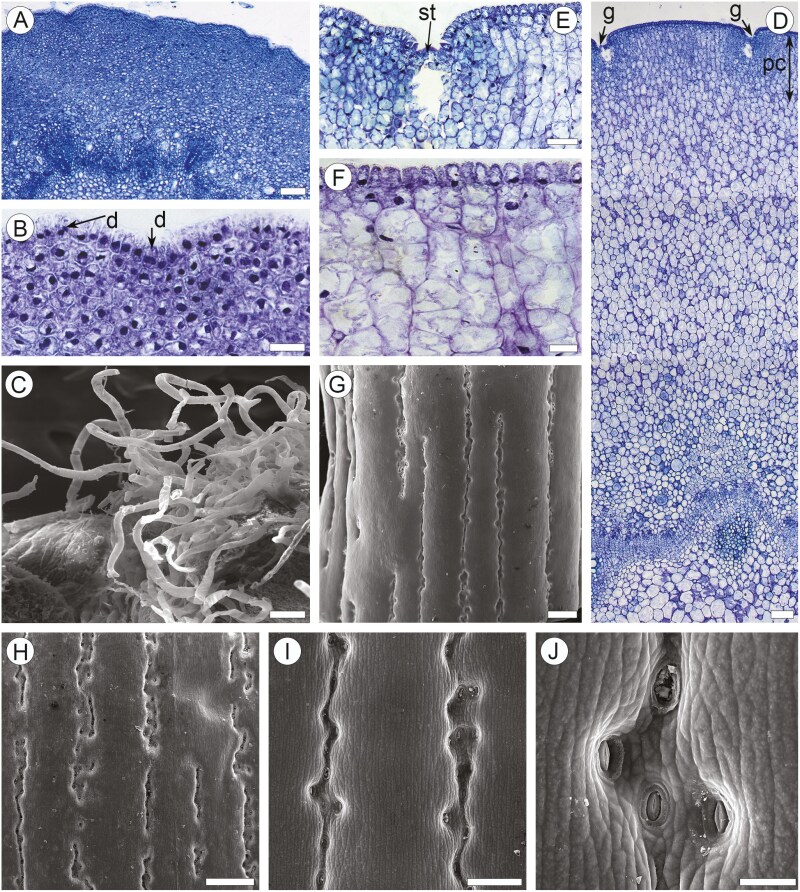
Anatomy of young stems of *Euphorbia tirucalli*. (a and b). TS of the stems of leaf-bearing shoots, light microscopy (LM). (a) Portion of entire stem: trichomes (t), homogenous cortex without palisade mesophyll; continuous ring of procambium with vascular bundles, pith. (b) Epidermis made of rectangular to vertically elongated papillar cells, lack of stomata, division of epidermal cells (d). (c) Trichomes on the stem epidermis of leaf-bearing shoot, SEM. (d–f) TS of the stems of leafless shoots (LM). (d) Portion of entire stem: lack of trichomes, stomatal grooves (g), palisade parenchyma in outer region of cortex, cambial zones without prominent rings of wood and secondary phloem, vascular bundle with primary phloem and xylem, pith. (e) Epidermis made of isodiametric cells, stomata (st) in grooves, stomatal chamber in palisade parenchyma. (f) Epidermis made of vertically elongated rectangular cells. (g–j) Surface of stem epidermis of leafless shoot with stomata in vertical grooves (SEM). Scale bars: a = 100 µm; b = 20 µm; c = 100 µm; d = 100 µm; e = 50 µm; f = 20 µm; g = 500 µm; h = 200 µm; i = 50 µm.

The stems bearing this bark are 2–4 mm in diameter. The epidermis is composed of a single layer of rectangular to vertically elongated bottle-like or papillar cells of 3–11 µm (up to 16 µm in [AO 768-24]) in tangential size (Table 1) and 15–22 µm in vertical size, with thin walls covered by thin cuticles as shown in (Fig. 2b). Anticlinal divisions occur in epidermal cells (Fig. 2b). Trichomes are abundant as shown in (Fig. 2a and c), non-glandular, multicellular with small basal cells. Stomata not found. Cortex with about 28–35 layers of isodiametric very thin-walled parenchyma cells of 6–14 µm in tangential size in the outer region of the cortex with 10–22 µm in its inner region, commonly containing chloroplasts. Narrow laticifers of 20–35 µm in diameter occur in the innermost region of the cortex. Crystals not found. The cortex is delineated from the pith by a nearly continuous ring of procambium, with vascular bundles of primary xylem and primary phloem. Pericyclic fibres not found.

### Juvenile bark on leafless twigs-shoots (Fig. 2d–j)

Stems bearing this bark are 4–13 mm in diameter (Fig. 2d). Epidermis comprises a single layer of vertically elongated rectangular cells to isodiametric thin-walled cells of 5–16 µm (up to 18 µm in [AO 768-24]) in tangential size (Table 1) covered by thin cuticle (Fig. 2e and f). No divisions observed in epidermal cells (Fig. 2f). Trichomes not found; their lapsed basal cells are indistinguishable from other epidermal cells. Stomata sunken, located in regular vertical grooves on the outer surface 20–50 µm in width (Fig. 2d, e, g–j), with prominent chambers beneath. The outer cortex is made of palisade parenchyma of about 0.2 mm in width, composed of 8–12 layers of thin-walled radially elongated parenchyma cells and radial strands of 2–5 cells, without prominent intercellular spaces (Fig. 2e–f). Clusters of smaller isodiametric cells (13–20 µm in diameter) associated with stomatal grooves occur in the outer cortex. The inner cortex is about 1.5 mm in width and consists of nearly isodiametric cells of 40–90 µm in tangential diameter, occasionally with small intercellular spaces (Fig. 2e). Laticifers of 55–65 µm in tangential diameter commonly occur in the inner cortex. Chloroplasts are present in the cells of the outer cortex. Crystalliferous cells were not found. The continuous ring of vascular cambium without prominent rings of secondary phloem and secondary xylem is found between the cortex and pith (Fig. 2d).

### Smooth mature bark on branches and stems (Fig. 3)

**Figure 3.**
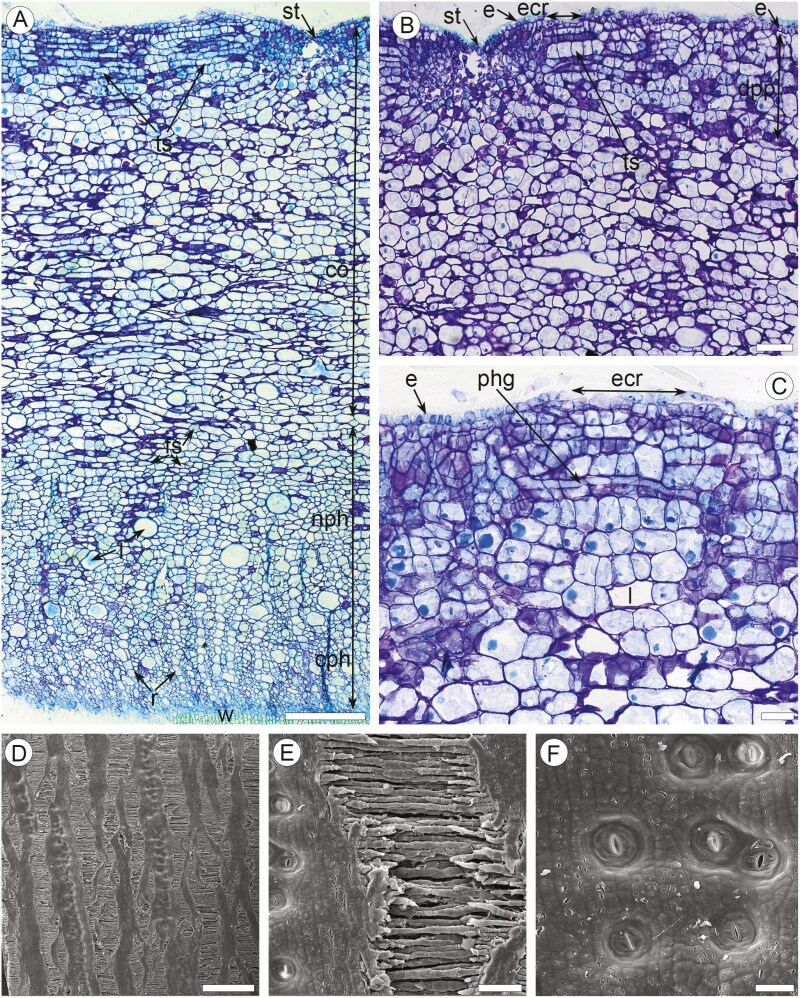
Anatomy of smooth mature bark on large stems of *Euphorbia tirucalli*. (a–c) TS of green mature bark (LM). (A) Entire bark: wood (w), conaducting (cph) and non-conducting (nph) secondary phloem with rays (r), laticifers (l) and fibre-sclereids (fs), dilated cortex (co), tangential strands (ts) of cortical parenchyma cells, stoma with stomatal chamber (st). (b) Outer region of bark: epidermis (e), stoma with stomatal chamber (st), expansion crack (ecr) with tangential bands of cortical parenchyma cells (ts), dilated palisade parenchyma (dpp). (c) Outer region of bark: epidermis (e), expansion crack (ecr) with tangential bands of cortical parenchyma cells, initiation of phellogen (phg) under the epidermal rupture, laticifer (l) in cortical parenchyma. (d–f) Surface of green mature bark (SEM). (d) Expansion cracks (ecr) and unfolded stomatal grooves (ugr) with stomata. (e) Expansion crack: tangential strands of cortical parenchyma cells. (f) Stomata on the surface of unfolded groove. Scale bars: a = 100 µm; b = 100 µm; c = 50 µm; d = 1 mm; e = 100 µm; f = 50 µm.

This stage was observed on the 34–61 mm stem diameter (Table 1). The epidermis persists at this stage, showing prominent dilatation traits. Epidermal cells are of the same shape and size as those on leafless shoots (Fig 3a–c). The stomatal grooves are unfolded, forming the vertical shallow depressions with superficial stomata (Fig. 3d–f). The portions of the epidermis between these zones are mostly ruptured, showing elongated vertical superficial ruptures ca. 150–300 µm in width. Dilatation of the cortical tissue is expressed by tangential stretching and anticlinal divisions of the cortical parenchyma cells, thus forming strands of 2–8 cells (Fig. 3b–e). Clusters of tightly arranged tangential cortical parenchyma strands are associated with epidermal ruptures (Fig. 3a–c, e). Tiny patches of resinous latex (recognized by yellow staining with Sudan IV similar in colour to the latex in laticifers) occur on the ruptured surfaces. The initiation of phellogen was observed in some of these clusters in the 6–7 cell layers beneath the epidermis (Fig. 3c). Chloroplasts were observed in the cells of the outer regions of dilated cortical parenchyma. Small solitary prismatic crystals occur in dilated cortical cells. The structure of the secondary phloem is the same as that of corky mature bark (see below). Laticifers are common in the dilated cortex and non-conducting secondary phloem. Fibre-sclereids of 35–42 µm in tangential size with very thick walls non-conducting secondary phloem.

### Contributions of epidermal stretching and expansion cracks to the increase in the circumference of juvenile and smooth mature bark (Fig. 4)

**Figure 4.**
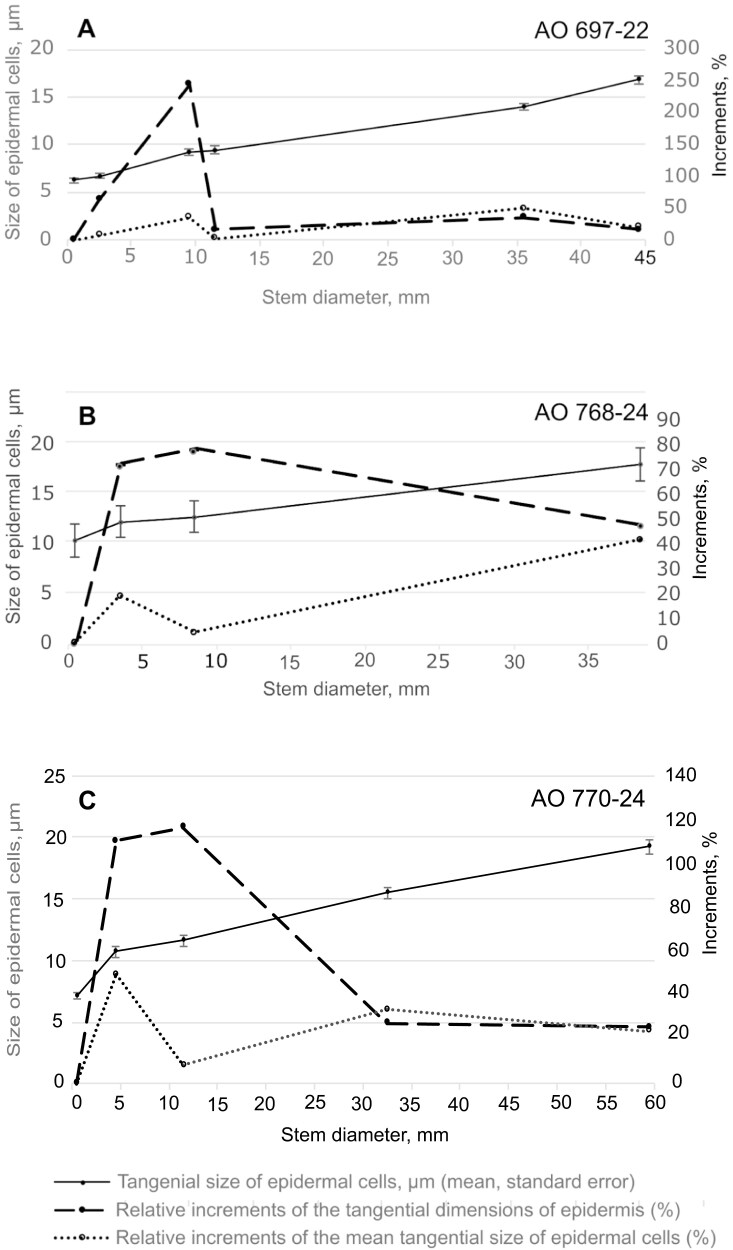
Mean tangential size of epidermal cells, its relative increments, as well as the relative increment of the tangential dimension of epidermis plotted against the stem diameter for three studied plant individuals of *Euphorbia tirucalli*. (a) Sample [AO 697-22]. (b) Sample [AO 768-24]. (c) Sample [AO 770-24]. The relative increments were calculated as percentages of the values of the same traits from the preceding stem portions of the same sample.

Table 1 and Fig. 4 present the quantitative data on the variation in tangential size of epidermal cells, the estimated tangential extensions of the epidermis, and of the expansion cracks in the smooth bark across a series of stem portions of varying diameters from three individuals of *Euphorbia tirucalli* (samples [AO 697-22], [AO 768-24], and [AO 770-24]). Both the size of the epidermal cells and the extension of the epidermis consistently increase as the stem girth expands, even after the formation of expansion cracks on the thickest portions (greater than 34 mm in diameter). The expansion cracks contribute between 42% and 72% of the bark girth in the examined samples (Table 1).

The relative increments of bark circumference in thinner stems (less than 12.5 mm in diameter, Fig. 4a), calculated as a percentage of the preceding stem portions of the same sample, are significantly greater (2.2 to 20.2 times) than the corresponding relative increments of the mean tangential size of epidermal cells. In thicker portions (greater than 12.5 mm in diameter), the relative increments in bark circumference or epidermis extension (in the portions with expansion cracks) are, however, nearly equal to the increments in the mean tangential size of epidermal cells (Table 1; Fig. 4).

These data suggest that the stretching of the epidermis in younger, thinner stems (up to about 12 mm in diameter) is insufficient to account for the observed increase in girth. It is highly likely that the increase in bark circumference in these stems is primarily due to anticlinal divisions of epidermal cells. It is probable that the meristematic activity of these cells ceases in thicker stems and that further increases in bark circumference in stems up to approximately 45 mm ([AO 697-22]; [AO 768-24]) or 62 mm in diameter ([AO 770-24]) are achieved solely through stretching and, later, also the formation of expansion cracks. Our data (Table 1; Fig. 4) confirm the sufficient contributions of these processes in achieving the observed epidermal extensions and expansion cracks in thicker stems. The increase in bark circumference on the stems with diameters greater than 45 mm 60 mm in ([AO 770-24]) is provided by the formation of periderm replacing the epidermis and outer regions of the cortex.

### Corky mature bark (Fig. 5)

**Figure 5.**
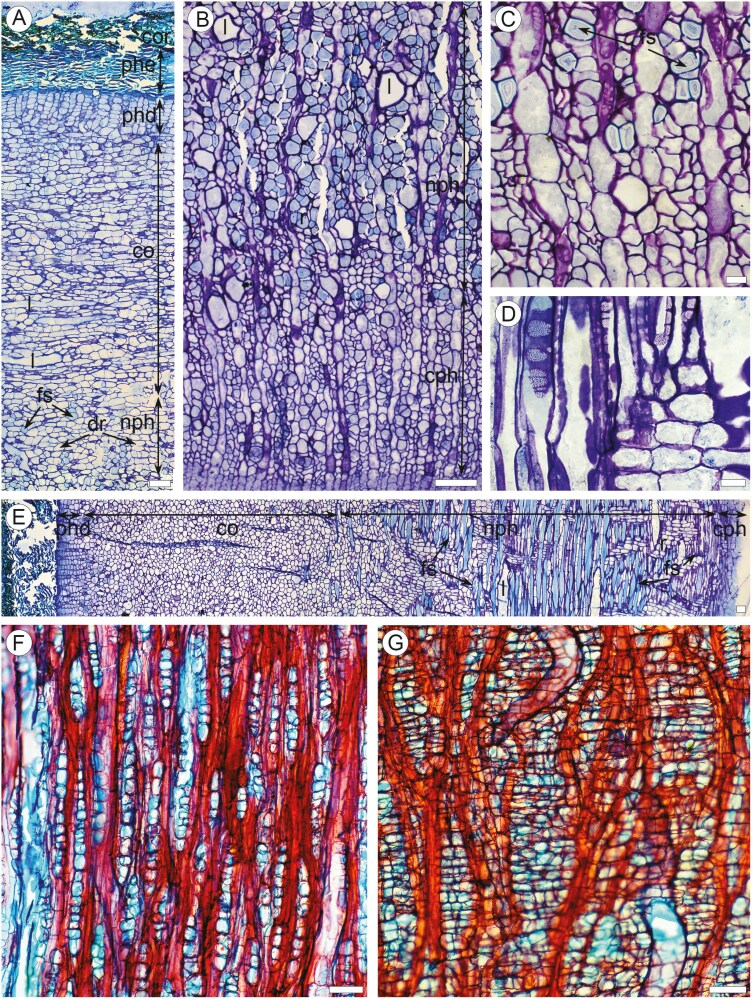
Anatomy of corky mature bark on large stems of *Euphorbia tirucalli* (LM). (a) TS of periderm, dilated cortex with an outer region of non-conducting secondary phloem (nph) with dilated rays (dr): remnants of abscised outer cortex (cor), phellem (phl), phelloderm (phd), laticifers (l) in dilated cortex. (b) TS of conducting (cph) and non-conducting (nph) secondary phloem with laticifers (l) and fibre-sclereids (fs). (c) TS of the transition zone between conducting and non-conducting secondary phloem: fibre-sclereids (fs) with thick multilayered walls. (d) Radial longitudinal section (RLS) of bark with conducting (cph) and non-conducting (nph) secondary phloem, cortex (co), phelloderm (phd), and phellem (phe): laticifers (l) and fibre-sclereids (fs) in non-conducting phloem; phloem rays made of procumbent and square cells. (e) RLS of conducting phloem: compound sieve plates. (f) Tangential radial sections (TRS) of inner region of non-conducting secondary phloem: mostly uniseriate phloem rays. (g TRS of outer region of non-conducting phloem: dilated 2–7-seriate phloem rays. Scale bars: a = 100 µm; b = 100 µm; c = 20 µm; d = 20 µm; e = 100 µm; f = 100 µm; g = 100 µm.

The appearance of cork was observed on stem portions of more than 45 mm in diameter ([AO 697-22], [AO 768-24]), or more than 62 mm in diameter ([AO 770-24]). In this stage, the bark is covered by a single continuous periderm, with remnants of the outer cortex on its surface (Fig. 5a). Phellem comprises 10–15 layers of radially flattened cells with thin inner walls, radial walls, and thick weakly suberized (Sudan IV test) outer walls. Dark tannin content occurs in the cells of the outermost phellem layers. Phelloderm is made up of 6–9 layers of radially flattened rectangular thin-walled cells. No crystalliferous cells and secretory cells are found in the periderm. The prominent dilated cortex of 0.7–0.9 mm in width is made mostly of tangential strands of 2–8 parenchyma cells that persist in mature bark.

The secondary phloem (Fig. 5b–e) is homogeneous and consists of conductive elements, axial and radial parenchyma, and non-articulated laticifers. Sieve tubes are 19–30 µm wide, solitary, and in radial or tangential groups of 2–4 (Fig. 5b). The sieve tube members are 387.4 µm in mean length (range 230–480 µm). Sieve plates are compound with 2–7 sieve areas located on vertical or slightly oblique cross walls (Fig. 5c). Axial parenchyma is in strands of 2–4 cells; crystals are not found. The transition from non-collapsed to collapsed secondary phloem is gradual, marked by the appearance of vertical laticifers and fibre-sclereids (Fig. 5b, c, and e). Fibre-sclereids in non-conducting secondary phloem are numerous, arranged into radial patterns, very thick-walled, 2104 µm in mean length (range 890–3660 µm), 22–38 µm in tangential size, with prominent multiple layers in cell walls (Fig. 5b, c, and e).

Dilation of the secondary phloem is affected mostly by tangential stretching and anticlinal divisions of ray parenchyma (Fig. 5a and b). Rays in non-dilated secondary phloem rays are uniseriate and composed of mostly square cells, occasionally with procumbent cells in their central portions (Fig. 5e and f). Dilated rays are enlarged by tangential expansion and anticlinal divisions up to 2–9-seriate ones (Fig. 5g). No crystals, sclereids, or secretory cells are found in ray parenchyma.

## Discussion

Our data on the structure of young stems of *E. tirucalli* are consistent with the results of other authors who examined the anatomy of this species (Rao *et al.* 1968, Kalashnik and Gaidarzhy 2013, Kalashnik 2015; Lekshmi *et al.* 2017) as well as of other members of *Euphorbia* (Mauseth, 2004; Thakur and Patil, 2012; Sultana, 2017; Acharya *et al*., 2021; James *et al*., 2022). Particularly, we confirm the lack of palisade chlorenchyma in the cortex of leaf-bearing shoots of *E. tirucalli* and its presence in leafless ones reported by Kalashnik and Gaidarzhy (2013) and Kalashnik (2015). Our observations show, however, that the leaves occur on all juvenile shoots of this species, and those fell off on older ones; no specialized leafless shoots were found. It is quite possible that the leaves on different shoots persist for different periods of time; testing this assumption is, however, beyond the scope of our study. Thus, we consider leaf-bearing and leafless shoots as subsequent stages of shoot development, but not as two different types of shoots, as Kalashnik and Gaidarzhy (2013) and Kalashnik (2015). Among other succulent species of *Euphorbia*, the gain of palisade chlorenchyma is associated with the reduction of leaves and, therefore, with the shift of photosynthetic functions to the stems (Kalashnik 2015). We observed this shift in the ontogeny of *E. tirucalli*: the transition from leaf-bearing shoots to leafless ones is contingent not only on the appearance of palisade parenchyma but also on the formation of stomata and stomatal grooves as well as on the loss of trichomes.

The stomata on leafless shoots of *E. tirucalli* are confined to long and deep vertical grooves (probably, these grooves also occur in *E. horrida* Boiss. as Mauseth’s (2004; Fig. 4H) microphoto suggests). The stomatal grooves are found in some taxa distributed in dry environments, for instance, on the stems of Casuarinaceae (Torrey and Howard Berg 1988) or the leaves of some Proteaceae (Jordan *et al.* 2008) and Asparagaceae (McClendon 1908, Johnson and Gale 1983). Such grooves were not reported in other stem succulents, but the deep crypts (areolar pits) containing stomata have been reported in the cactus *Blossfeldia liliputana* Werderm. (Barthlott and Porembski 1996). The functions of these grooves, crypts, and other epidermal depressions separating stomatal pores from the atmosphere are not fully understood: these structures are considered as the tools to reduce water loss, to facilitate the CO_2_ access into thick leaves, to protect stomata against environmental stress factors or pathogens, or to improve hydraulic safety by creation of humid microenvironment (Šantrůček 2022). As *E. tirucalli* and *E. horrida* use the CAM photosynthetic pathway (Horn *et al.* 2014), it is likely that the facilitation of the CO_2_ supply into the cortical parenchyma of succulent stems for its accumulation during the night-time (Hassiotou *et al.* 2009) is the main benefit of stomatal grooves in these species.

Smooth green bark is found not only on young shoots of *E. tirucalli* but also on its thick branches and trunk up to 6 cm in diameter. The transformations of this bark in the course of dilatation are noteworthy: its stomatal grooves are stretched out, bringing stomata to the surface, whereas the epidermis between grooves is ruptured, forming broad vertical cracks underlaid by tightly arranged tangential strands of cortical parenchyma. Such ruptures show great resemblance in the pattern of their formation to the expansion cracks described by Schneider (1955) on the bark of young shoots of lemon trees (*Citrus* × *limon* (L.) Osbeck). These cracks also appear as epidermal breakings associated with the clusters of tangentially stretched cortical parenchyma, which are the sites of subsequent phellogen initiation. In the lemon tree, however, the periderm arises in these clusters shortly after rupturing the epidermis. Unlike that, the prominent ruptures found on the smooth mature bark of *E. tirucalli* mostly lack the periderm, which is obviously formed sometime after their emergence and, therefore, is not involved in bark dilatation at this stage. In that respect, these ruptures resemble the experimental stem injuries both in *E. tirucalli* (Anandan *et al*., 2018) and in other plant taxa (Borger and Kozlovski 1972, Esau 1977, Wier *et al.* 1996, Anandan *et al.* 2018) showing the late development of wound periderm appeared in more than seven days after wounding. Unlike such injuries, however, the ruptures on the smooth mature bark of *E. tirucalli* do not show any traits of the sealing of their exposed surface either by necrosed cells (Esau 1977) or by permanent latex plugs, which are typical for wound response of the species under study (Anandan *et al.* 2018). These ruptures make the most significant contribution to the tangential expansion of smooth mature bark before forming the periderm: they account for 42% to 73% of the stem circumference (Table 1).

Thus, we may consider the epidermal ruptures on the smooth mature bark of *E. tirucalli* as a kind of expansion cracks *sensu*Schneider (1955) with the delayed formation of the periderm. We did not find any other publications than Schneider’s (1955) article confirming the occurrence of expansion cracks in other plant groups, including stem succulents. To all appearances, these cracks occur more commonly within different plant taxa than is generally appreciated, at least in the bark of young shoots. Still, these ruptures are improperly taken for lenticels, the structures of peridermal origin. Their resemblance to lenticels suggests that the expansion cracks may also facilitate gas exchange between the environment and the inner bark. This physiological role is considered particularly important in photosynthesizing barks in stem succulents and non-succulent plants. Obviously, the occurrence of these structures and their role in bark dilatation deserve consideration by plant anatomists.

The deep initiation of phellogen found in *E. tirucalli* is not characteristic of the entire genus. While this condition has also been reported *E. characias* L. and probably in *E. esula* L. (Schweingruber and Landolt 2005), the subepidermal phellogen initiation found in *E. milii* Des Moul. (Kalashnik and Gaidarzhy 2013), *E. hierosolymitana* Boiss. (Crivellaro *et al.* 2013), *E. balsamifera* Aiton and *E. cyparissias* L. (Schweingruber and Landolt 2005). Unlike *Euphorbia*, the stem succulents from other groups studied to date, i.e. Cactaceae (Gibson and Nobel 1986, Mauseth 2006, Garcia *et al.* 2012) and succulent *Senecio*, Asteraceae (Mauseth 2004) share epidermal initiation of the periderm. Generally, the occurrence and development of periderm on succulent photosynthetic stems need more comprehensive exploration.

The bark on the oldest parts of the trunk of *E. triucalli* is covered with a single ring of periderm, which has a relatively thick, non-stratified phellem and prominent phelloderm. The dilated cortex persists in this bark, but we did not observe any traits of subsequent periderms. Such allocation of the tissues suggests the ability of the periderm to continuously stretch tangentially with the formation of shallow fissures in the outer region of its phellem. The cortex also maintains the continuity of bark, serving as a dilatation tissue (Whitmore 1962, Kotina *et al.* 2012, 2017, Shtein *et al.* 2023). Among other *Euphorbia*, the structure of mature periderm has been described only in *E. espinosa* Pax, a leaf-bearing succulent shrub from eastern Africa (Mtombeni 1994). Unlike *E. tirucalli*, this species has papery peeling bark (Coates Palgrave 2002) with reticulate rhytidome (Mtombeni 1994). The allocation of separation layers in its peeling bark was not reported by Mtombeni (1994).

Our observations on the bark structure of *E. tirucalli* are mostly consistent with Mtombeni’s (1994) anatomical description of this species. However, while Mtombeni (1994) described the sclerenchyma cells in the bark of this species as fibres, we prefer to use the term ‘fibre-sclereids’ for them following the recommendations of the IAWA Committee (Angyalossy *et al.* 2016). Obviously, these cells are similar to the fibres in their shape and significant length (up to 3.7 mm), manifesting a prominent intrusive elongation during their differentiation. Their localization in the dilated cortex and non-conducting secondary phloem (coupled with their lack in the conductive one) strongly suggests that those cells are derived from axial or cortical parenchyma cells. In contrast with them, the phloem fibres develop either from procambium (primary phloem fibres) or from fusiform cambial derivatives (secondary phloem fibres). Apart from their origin from parenchyma, the long sclerenchyma cells in *E. triucalli* are similar to typical sclereids also found in other Euphorbiaceae by Roth (2005) in having poly lamellate cell walls. Thus, these cells strictly correspond to the definition of fibre-sclereids as ‘elongated sclereids with characteristics intermediate between those of a fibre and a sclereid’ (Angyalossy *et al.* 2016). Among other Euphorbiaceae, the ‘fibres’ reported in the bark of *Croton draco* Schltdl. (Farías *et al.* 2009) are similar to the fibre-sclereids of *E. tirucalli* in distribution and wall structure and must be termed in the same way. The types of sclerenchyma cells in the bark of other family members are described as ‘fibres’ (Zahur 1959, Mtombeni 1994, Roth 2005) and require careful reconsideration. To date, we have not found any reliable evidence for the occurrence of secondary phloem fibres derived from vascular cambium in any members of Euphorbiaceae, but we found those in a closely related family, Picrodendraceae (Maruta and Oskolski 2021).

## Conclusion

Stomata and stomatal grooves on the stem epidermis of *E. tirucalli* are formed after shedding the leaves. Facilitation of the CO_2_ supply into the cortical parenchyma is thought to be the main functional benefit of stomatal grooves. The stomatal grooves are stretched out during the bark dilatation, bringing stomata to the surface.

Epidermis persists on thick branches and trunks of *E. tirucalli*, but it is dilated not only by stretching of epidermal cells but mostly by the formation of expansion cracks, i.e. broad vertical epidermal ruptures underlaid by tightly arranged tangential strands of cortical parenchyma. The periderm is not involved in the bark dilatation of that stage, as it is initiated beneath the epidermal ruptures long after their formation. This way of bark dilatation has not been reported elsewhere.

The initiation of phellogen in the depth of the cortex found in *E. tirucalli* is not common in other *Euphorbia* and has not been reported in other stem succulents.

Sclerenchyma cells in the bark of *E. tirucalli* must be considered fibre-sclereids rather than fibres.

## Data Availability

The data underlying this article are available in the article.
